# Prognostic Value and Immune Landscapes of Four Types of RNA Modification Writer-Related LncRNAs Signature in Lung Adenocarcinoma

**DOI:** 10.7150/jca.96755

**Published:** 2024-07-09

**Authors:** Yongmei Qian, Qicheng Zhang, Yinghui Ren, Limin Cao, Sijia Zheng, Bingbing Li, Xiang Wu, Zhaowei Meng, Ke Xu

**Affiliations:** 1 Tianjin Key Laboratory of Lung Cancer Metastasis and Tumor Microenvironment, Tianjin Lung Cancer Institute, Tianjin Medical University General Hospital, Tianjin 300052, China.; 2 Department of Anesthesiology, Tianjin First Central Hospital, Tianjin 300192, China.; 3 Core Facility Center, Tianjin Medical University General Hospital, Tianjin 300052, China.; 4 Department of Nuclear Medicine, Tianjin Medical University General Hospital, Tianjin 300052, China.

**Keywords:** lung adenocarcinoma, RNA modification writer, long non-coding RNA, prognosis, immunotherapy, tumor microenvironment

## Abstract

**Background:** Lung adenocarcinoma (LUAD) is the predominant pathological subtype of non-small cell lung cancer (NSCLC). The four primary forms of RNA adenosine modifications, N6-methyladenosine (m^6^A), N1-methyladenosine (m^1^A), alternative polyadenylation (APA) and adenosine-to-inosine (A-to-I) RNA editing, play a critical role in tumor progression. However, the clinical significance of RNA modification writer-related long non-coding RNAs (lncRNAs) in LUAD remains unclear.

**Methods:** The Cancer Genome Atlas (TCGA) database was used to obtain transcriptomic and clinicopathological data. Univariate Cox regression analysis, consensus cluster analysis, and least absolute shrinkage and selection operator (LASSO) Cox regression were used to establish the molecular subtypes and prognostic signatures of LUAD based on the expression levels of lncRNAs. ESTIMATE, CIBERSORT, ssGSEA, and TIDE algorithms were used to investigate immune cell infiltration and immunotherapy. In addition, IC_50_ of chemotherapeutic agents were calculated for different risk subgroups using the "pRRophetic" R package. Finally, the expression of prognosis-associated lncRNAs in lung cancer tissues was verified using qPCR.

**Results:** A prognostic risk signature containing seven lncRNAs associated with four types of RNA modification writers was established. The high-risk group had a poorer prognosis and higher clinicopathological grade. Most immune checkpoint genes and immune cell infiltration differed significantly between the two risk groups. The high-risk group had a higher tumor mutation burden (TMB), lower TIDE score, and was more sensitive to immunotherapy.

**Conclusion:** We developed an RNA modification writer-related seven-lncRNA signature prognostic model that was associated with prognosis, tumor microenvironment, and response to immunotherapy in LUAD patients. Among them, LINC01352, AC024075.1, AC005070.3, AL133445.2, AC005856.1, and LINC00968 were downregulated in LUAD, whereas AC092168.2 was upregulated. This model may be a valuable tool for personalized LUAD therapies.

## 1. Introduction

Lung cancer is a common cause of cancer-related deaths 1. Approximately 85% of lung cancers are non-small cell lung cancer (NSCLC), and lung adenocarcinoma (LUAD) is a major subtype of NSCLC 2. Despite considerable achievements in cancer treatment, lung cancer remains one of the most aggressive and fatal tumor types, with a 5-year survival rate of less than 15% 3. Therefore, a precise prognostic model, based on epigenetic features, is required.

RNA modification is a novel form of post-transcriptional regulation. More than 100 types of epigenetic modification are present in RNA 4, including mRNA, tRNA, rRNA, lncRNA, small nuclear RNA (snRNA), and microRNA (miRNA) 5. RNA modifications regulate RNA metabolism, including RNA processing, splicing, nuclear export, transcript stability and translation efficiency 6. Dysregulation of RNA modification is involved in tumor progression, suggesting that RNA modification could be used for tumor prognosis 4.

Among the various RNA modifications, adenosine is the most heavily modified nucleotide, and m^6^A, m^1^A, APA, and A-to-I are the commonly studied adenylate modifications 7, 8. M^6^A modification is a methylation that occurs at the sixth nitrogen position of adenosine. This modification is catalyzed by methyltransferases, so called “writers,” including METTL3, METTL14, METTL16, VIRMA, and WTAP. M^6^A modification affects the expression of oncogenes or tumor suppressors and plays important roles in many cancer types, such as tumors of the prostate 9, breast 10, and lungs 11. The M^1^A modification is performed by writers such as TRMT61A, TRMT61B, TRMT10C, and TRMT6 12. M^1^A modification, responsible for RNA stability and translation efficiency, promotes tumor progression by regulating gene expression and signal transduction 13, 14. APA is regulated by CPSF, CSTF, CFI, CFII, NUDT21, and the PABPN1 protein complex 15; A-to-I is mediated by ADARs 16. APA and A-to-I are also involved in tumorigenesis. Notably, cross-talks between different RNA modifications have been reported. Molinie *et al.* showed that the presence or absence of m^6^A modifications in transcripts results in different distributions of APA modification sites 17. Xiang *et al.* found a negative relationship between m^6^A and A-to-I 18. RNA modification writers can modulate tumor immunity, which may provide a new strategy for immunotherapy of tumors 19.

LncRNAs are transcripts over 200 nucleotides in length with limited or no protein-coding roles. The lncRNAs regulate gene expression, and influence cellular homeostasis, including proliferation, survival, migration, and genomic stability, and play important roles in tumorigenesis and tumor progression. The association between RNA modifications and lncRNAs in cancer has been demonstrated. Wen *et al.* found that high m^6^A levels in the lncRNA NEAT1-1 were associated with bone metastasis in prostate cancer 9. Xue *et al.* showed that METTL3-catalyzed m^6^A modification in lncRNA ABHD11-AS1 promotes the Warburg effect in NSCLC 20. Interestingly, lncRNAs also regulate RNA modification. Zhu *et al.* reported that the peptide produced by LINC00266-1 interacts with the m^6^A reader IGF2BP1 to increase mRNA stability and c-Myc expression, thereby promoting tumorigenesis 21.

To understand the roles of RNA modification writer-related lncRNAs in the prognosis and immune response of LUAD, we systematically profiled four types of RNA modification (m^6^A, m^1^A, A-to-I, and APA) writer-related lncRNAs in LUAD for the first time. We classified LUAD patients into two subgroups (clusters 1 and 2) with distinct clinicopathological features and immune microenvironment infiltrative profiles. Furthermore, we constructed a prognostic signature consisting of seven RNA modification writer-related lncRNAs, which may help improve the treatment of LUAD patients in the future.

## 2. Materials and Methods

### 2.1 Data collection and processing

Data on RNA sequencing and mutation and matching clinical information for the TCGA-LUAD cohort were obtained from the Genomic Data Commons (GDC) Data Portal (https://portal.gdc.cancer.gov/), which contains information on 504 LUAD and 58 normal specimens. In addition, we downloaded somatic copy number variation (CNV) data from the UCSC Xena database (http://xena.ucsc.edu/) and obtained gene annotation files from GENCODE (https://www.gencodegenes.org/human/). Immunological subtype files, stemness score (DNA methylation-based) files, and stemness score (RNA expression-based) files were downloaded from the TCGA Pan-Cancer (PANCAN) database.

We extracted expression profile data of four types of RNA modification writers, including eight m^6^A modification enzymes (METTL3, METTL14, METTL16, WTAP, KIAA1249, RBM15, RBM15B, and ZC3H13), six m^1^A modification enzymes (TRMT6, TRMT61A, TRMT61B, TRMT10C, BMT2, and RRP8), twelve APA modification enzymes (PCF11, CFI, CLP1, NUDT21, PABPN1, CPSF1/2/3/4, and CSTF1/2/3), and three A-to-I modification enzymes (ADAR, ADARB1 and ADARB2). Of these, we excluded genes with missing sequencing data in the TCGA database. In addition, lncRNA sequencing data were extracted for subsequent analyses. We excluded genes with low expression (average FPKM values below 0.1 in all samples).

### 2.2 Mutation analysis and expression of RNA modification writer analysis

We analyzed the mutation data using the “maftools” package and plotted the waterfall for the writers of RNA modification in LUAD patients. Moreover, we analyzed CNV in LUAD patients and used the “RCircos” package to generate CNV changed locations of RNA modification writers on 23 chromosomes. The “limma” (R package) was used to identify RNA modification writers differentially expressed in LUAD in the TCGA-LUAD cohort, and the “pheatmap” (R package) was used for visualization.

### 2.3 Co-expression analysis and identification of the prognostic value of m^6^A/m^1^A/APA/A-to-I writer-related lncRNAs

Pearson correlation analysis was applied to filter four types of RNA modification writer-related lncRNAs by setting |R| >0.5 and p <0.001. Univariate Cox regression analysis was performed to identify four types of RNA modification writer-related lncRNAs with prognostic values using a criterion of p <0.01. The co-expression network was plotted by the “igraph” (R package). The expression differences of RNA modification writer-related lncRNAs associated with prognostic value between tumor and normal tissues visualized with the “pheatmap” and “ggpubr” (R packages) and were assessed by the Wilcoxon test.

### 2.4 Consensus clustering of RNA modification writer-related lncRNAs

Based on the expression of RNA modification writer-related lncRNAs with prognostic significance, the "ConsensusClusterPlus" (R package) was used to perform unsupervised consensus clustering on 504 LUAD patients into potential molecular subtypes (The clinical information of the two groups of patients is presented in Supplementary Table 1) 22. To determine if these samples could be visually distinguishable, principal component analysis (PCA) and t-distributed stochastic neighbor embedding (T-SNE) analysis were performed by using the “ggplot” and “Rtsne” (R packages) 23, 24. The Kaplan-Meier (K-M) curve was used to compare the overall survival (OS) rate between different clusters using the log-rank test with “survival” and “survminer” (R packages). Clinical information was incorporated to analyze different clinicopathological characteristics of different molecular subtypes by using the “pheatmap” (R package). Gene set enrichment analysis (GSEA) was then used to identify distinct pathways of functional enrichment in samples from distinct clusters. Moreover, the CIBERSORT Algorithm was applied to assess the proportion of 22 types of tumor-infiltrating immune cells (TICs) for each sample 25, and the "limma" (R package) proportion was utilized to screen for discrepancies in immune cell infiltration of patients in different subgroups. To investigate the features of the TME in different subtypes of patients, TME scores (including immune, stromal, and ESTIMATE scores) were calculated using the ESTIMATE algorithm 26. In addition, we explored the expression of 28 immune checkpoint genes in different subtypes 27.

### 2.5 Construction and validation of RNA modification writer-related lncRNAs signature

The TCGA-LUAD cohort was randomly divided into two sets in a 1:1 ratio, named the training and test sets, for risk model construction and validation, respectively. No statistically significant differences were observed between the two cohorts in terms of clinicopathological characteristics (Supplementary Table 2). The total TCGA-LUAD cohort was defined as the “entire set.” The least absolute shrinkage and selection operator (LASSO) Cox regression analysis 28 was applied to further shrink the range of potential lncRNAs and build up the lncRNAs signature associated with RNA modification writers with the “glmnet” and “survival” (R packages). The following equation was used to derive the risk score: Risk score = Σ (βi × Expi) (β: coefficients; Exp: FPKM value of each m^6^A/m^1^A/APA/A-to-I -related lncRNA). Subsequently, the K-M curves were plotted with the R package “survival” and “survminer” to assess the availability of the prognostic model, and the receiver operating characteristic (ROC) curves were created with the R package “timeROC” to evaluate the prognostic sensitivity and accuracy of the signature construction. Univariate and multivariate regression were performed to evaluate whether the risk score in both cohorts could serve as an indicator of independent prognosis. The survival status of both risk subgroups was assessed in the training subset, and the outcomes were validated in the test subset. To explore the potential differences between the high- and low-risk subgroups, PCA analysis was performed with the R package "scatterplot3d.”

### 2.6 Prognostic value and clinical correlation analysis of risk scores

To further elucidate the prognostic value of the risk scores, we performed survival analysis of patients with different clinicopathological characteristics, including sex (male, female), grade (I-II, III-IV), T stage (T1-2, T3-4), N stage (N0, N1-2) and M stage (M0, M1). Heatmaps were created to assess the clinical characteristics of high- and low-risk subgroups. Differences in the risk scores of patients with different clinicopathological features were analyzed using “ggpubr” (R package). Furthermore, Gene Ontology (GO) and Kyoto Encyclopedia of Genes and Genomes (KEGG) analyses were performed.

### 2.7 Immune landscape evaluation of the risk scores

As previously described, the CIBERSORT Algorithm was performed to value the proportion of 22 types of TICs in each tumor sample, and the R package “limma” was applied to screen the differences in immune cell infiltration of patients with varying risk scores. The correlation between the risk scores and immune cell infiltration was assessed using the Spearman correlation test. In addition, we used seven algorithms (xCELL, Timer, Quantiseq, MCPcounter, EPIC, CIBERSORT-ABS, and CIBERSORT) to systematically analyze and demonstrate the relationship between risk scores and tumor microenvironmental cell infiltration. Subsequently, the expression of immune checkpoint genes in different risk subgroups was investigated. Immune function-related datasets were acquired from the MSigDB database (https://www.gsea-msigdb.org/gsea/index.jsp) and analyzed using the “GSVA” (R package).

### 2.8 Mutation analysis and evaluation of response to antitumor therapy

Genes with remarkable mutations in both risk subgroups were assessed and visualized using “maftools” (R package). Differences in tumor mutation burden (TMB) across risk subgroups were assessed using the Wilcoxon rank-sum test. The tumor immune dysfunction and exclusion (TIDE) algorithm 29 (http://tide.dfci.harvard.edu) was used to predict the possibility of an immunotherapy response. To predict the sensitivity of immunotherapy, we downloaded the immunophenoscores (IPS) of LUAD patients from the TCIA database (https:// tcia. at home) to compare the differences in IPS between the high- and low-risk groups receiving different immunotherapy regimens. Furthermore, we downloaded data from the Genomics of Drug Sensitivity in Cancer (GDSC) database to predict the reactions of LUAD patients to common antineoplastic drugs. The “pRRophetic” (R packages) was performed to calculate the half-maximal inhibitory concentration (IC_50_) of chemotherapy drugs in different risk subgroups 30.

### 2.9 Prognosis and tumor microenvironment correlation analysis of single lncRNA

Samples from LUAD patients were divided into high- and low-expression groups based on the median expression of individual lncRNAs in the prognostic signature of RNA modification writer-related lncRNAs. The R package “survival” was used to obtain the survival profiles of the individual lncRNAs. Subsequently, we analyzed the differential expression of individual lncRNAs in the different immune subtypes. We further evaluated the correlation of seven m^6^A/m^1^A/APA/A-to-I-related lncRNAs with the TME score calculated using the ESTIMATE algorithm and stemness index (including RNA stemness scores [RNAss] and DNA stemness scores [DNAss]).

### 2.10 qPCR

qPCR was conducted to detect lncRNA expression levels. RNA was isolated from the tissues of lung cancer patients using trizol (Invitrogen), and reverse transcription was performed using a Takara kit (Dalian, China). LncRNA expression was assessed by qPCR using the Power SYBR Green Master Mix (Thermo Fisher Scientific). Primer sequences are listed in Supplementary Table 3. GAPDH was used for normalization. This study was approved by the Ethics Committee of TMUGH.

### 2.11 Statistical Analysis

The R language software (version 4.2.1) (http://www.r-project.org/) was used to analyze and visualize the data. *P* <0.05 was considered statistically significance, unless otherwise stated.

## 3. Results

### 3.1 Gene mutation landscape and expression of four types of RNA modification writers in LUAD

**Figure 1:**
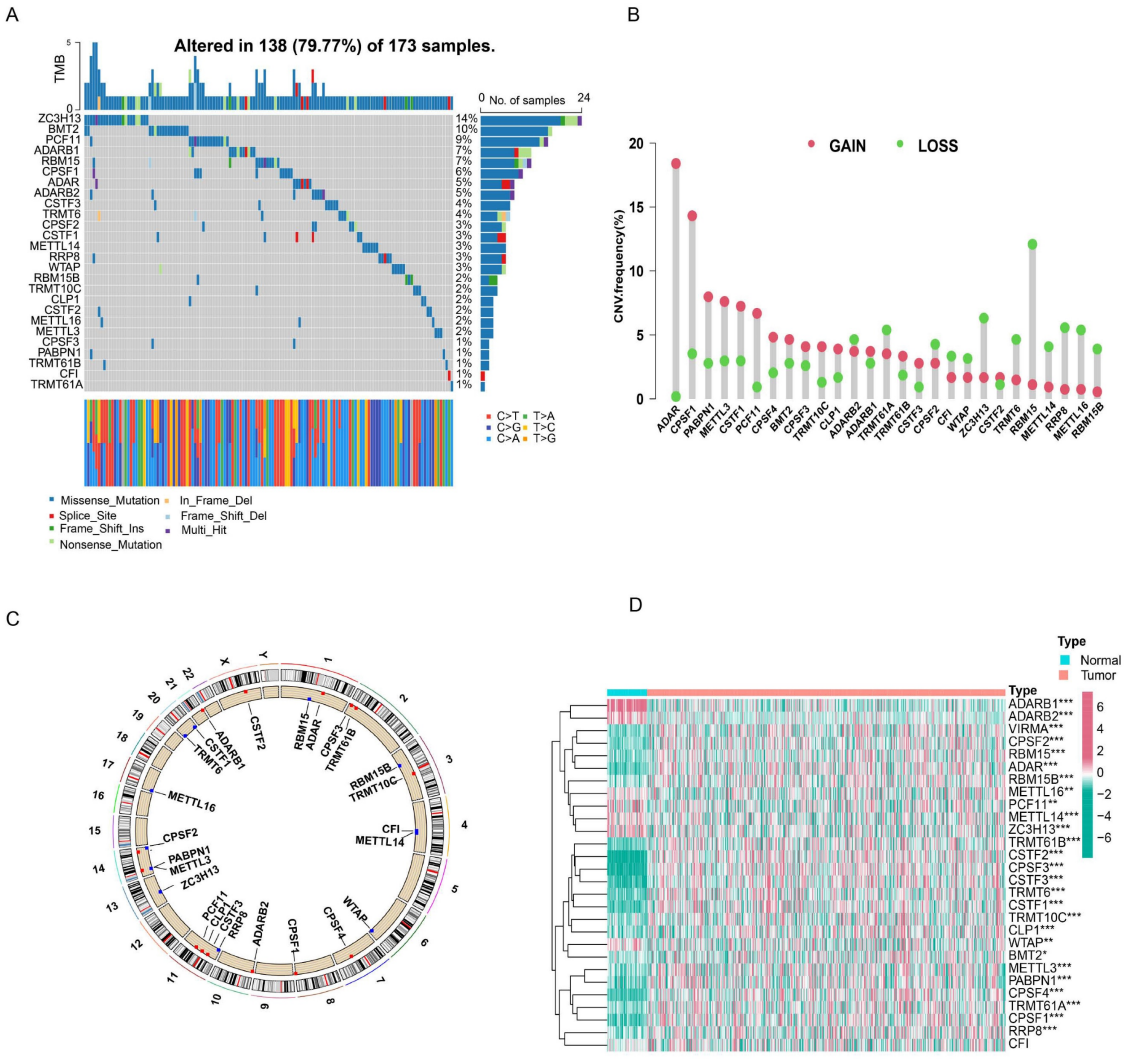
**Gene mutation landscape and expression of four types of RNA modification writers in LUAD. (A)** Mutation waterfall in lung adenocarcinoma patients from the TCGA-LUAD cohort. **(B)** CNV frequency of RNA modification writers in the TCGA-LUAD cohort. **(C)** The location of CNV alterations for RNA modification writers on chromosomes in the TCGA-LUAD cohort. **(D)** The heatmap of differential expression of RNA modification writers between normal (n = 58) and lung adenocarcinoma tissues (n = 504) in the TCGA-LUAD cohort.

First, we investigated the occurrence of somatic mutations in 29 RNA modification writers from the TCGA-LUAD database. Of the 173 samples, 138 (79.77%) exhibited genetic mutations in RNA modification writers. Figure 1A summarizes the top 26 mutant genes. Among these, ZC3H13 (14%) was the most frequently mutated gene, followed by DMT2 (10%) and PCF11 (9%). Moreover, all RNA modification writers had prevalent CNV alterations (Figure 1B). Among these, ADAR, CPSF1, and PABPN1 showed remarkable copy number amplification, whereas ZC3H13, RBM15, and METTL14 showed significant copy number depletion. The locations of CNV changes in these genes on the chromosome are shown in Figure 1C.

Next, we examined the expression of m^6^A/m^1^A/A-to-I/APA writers in TCGA-LUAD samples. As shown in Figure 1D, 27 RNA modification writers were differentially expressed in LUAD tissues. Among these genes, 20 genes were upregulated in LUAD tissues, whereas 7 genes were downregulated in LUAD tissues. These data indicated that RNA modification writers are highly heterogeneous in terms of genetic variation and expression, suggesting that they may play important roles in the initiation and progression of LUAD.

### 3.2 m^6^A/m^1^A/A-to-I/APA modification writers-related lncRNAs

**Figure 2:**
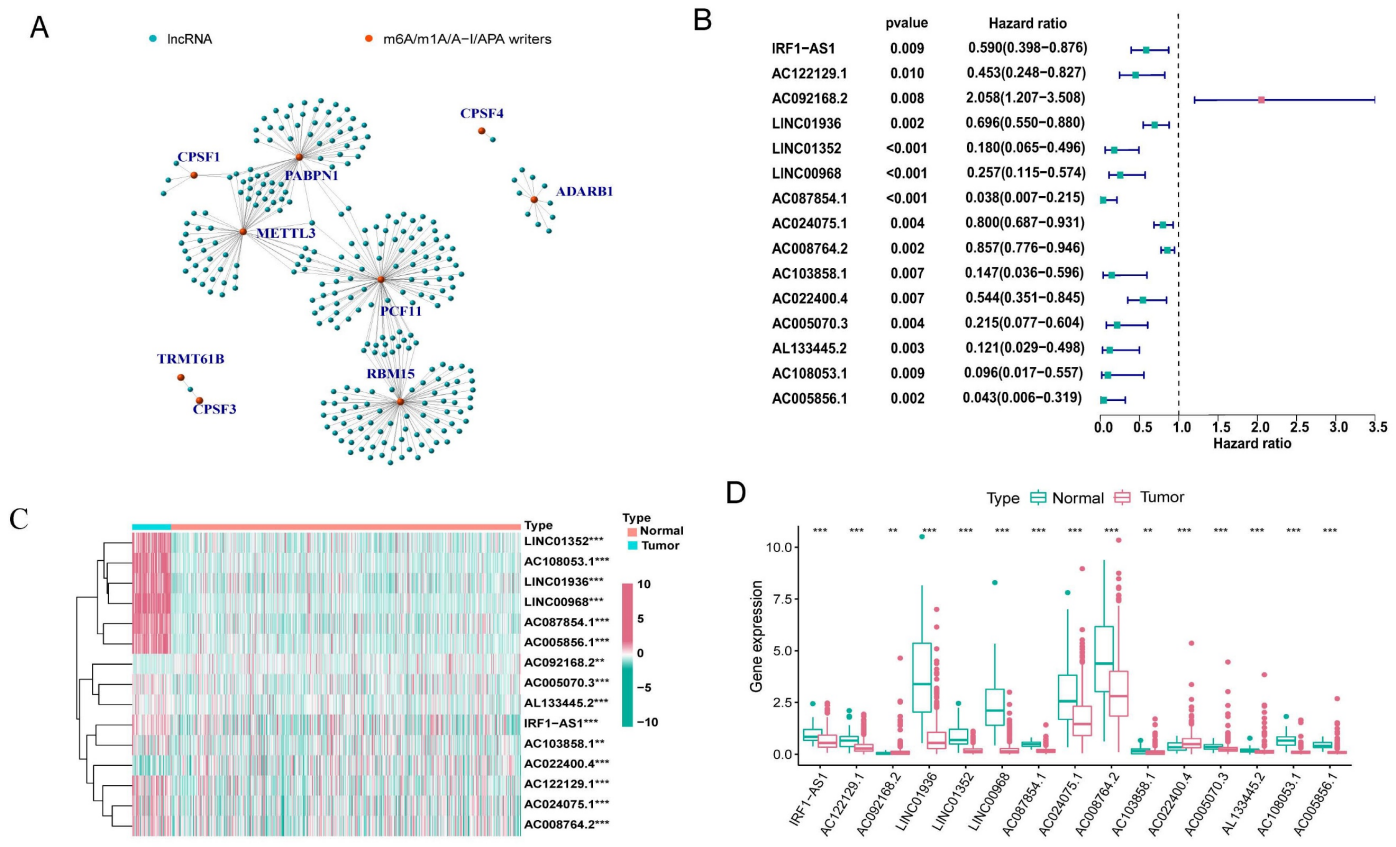
**Identification of m^6^A/m^1^A/A-I/APA modification writers-related lncRNAs. (A)** 269 lncRNAs co-expressed with m^6^A/m^1^A/A-I/APA modification writers. Green represented lncRNAs, while red represented m^6^A/m^1^A/A-I/APA modification writers. **(B)** The forest plot showing the 15 lncRNAs with prognostic value screened by univariate Cox regression analysis. **(C, D)** The heatmap and box plot of differential expression of 15 lncRNAs with prognostic value between normal (n = 58) and tumor samples (n = 504) in the TCGA-LUAD cohort.

The expression matrix of 10,233 lncRNAs was extracted from our transcriptome FPKM data, which were obtained and sorted from the TCGA database. Using Pearson correlation analysis with filtering standards of | Pearson R| > 0.5 and p-value < 0.001, we screened 269 lncRNAs that were co-expressed with four RNA modification writers (m^6^A, m^1^A, A-to-I, and APA) (Figure 2A). We further analyzed 15 lncRNAs with potential prognostic significance from the 269 co-expressed lncRNAs using Cox regression analysis (p < 0.01). Forest plots indicated that 14 lncRNAs had a protective effect with a hazard ratio (HR) < 1, except for AC092168.2 which was a risk factor with HR > 1 (Figure 2B). The heatmap and box plot showed that the expression of 15 lncRNAs differed significantly between normal and LUAD samples, and that most of them were expressed in tumor tissues at a lower level than in normal samples (Figure 2C, D). These results suggested that most of the 15 lncRNAs acted as protective factors.

### 3.3 Consensus clustering of RNA modification writers-related lncRNAs identified two clusters of LUAD patients

**Figure 3:**
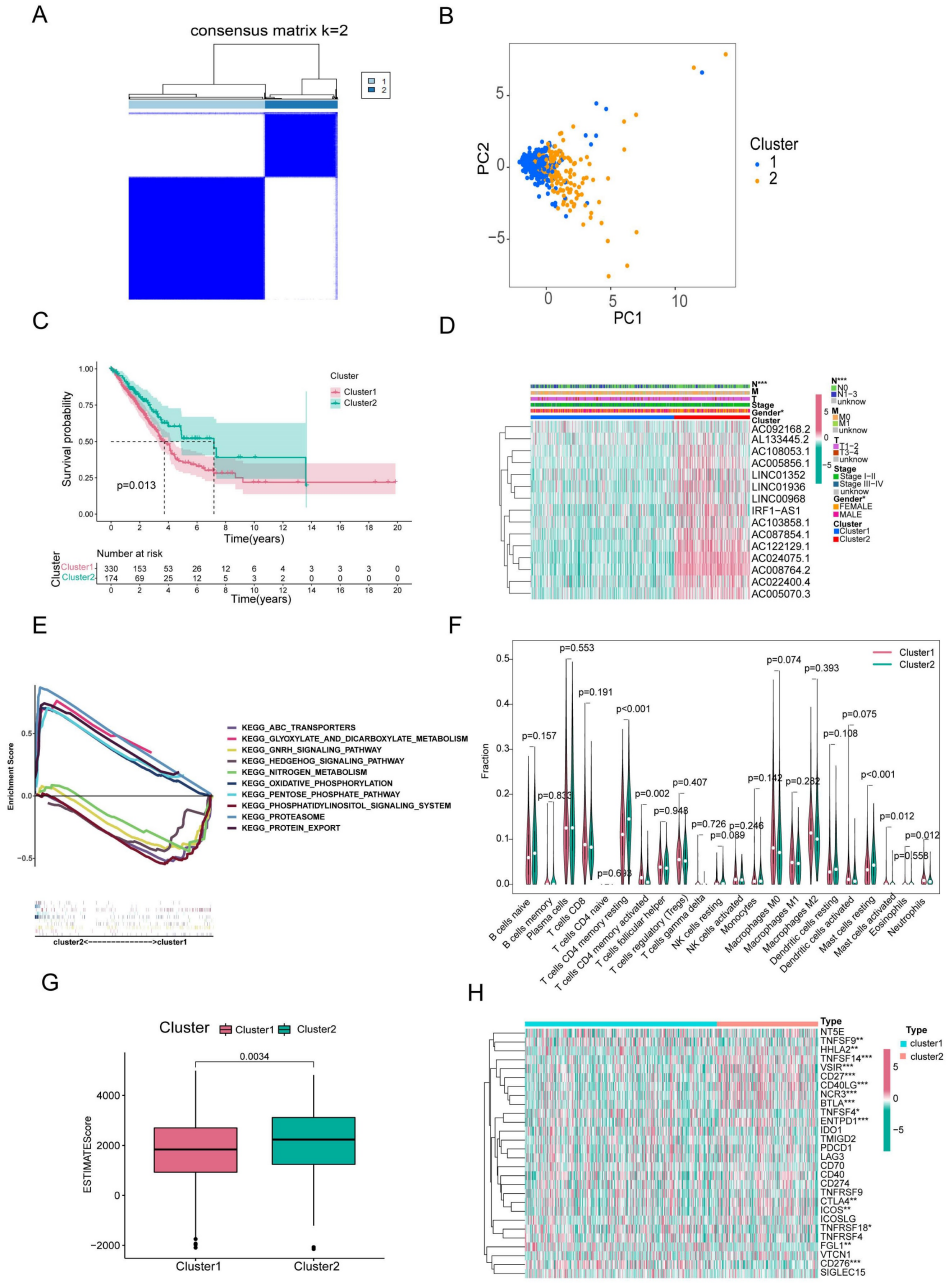
** Overall survival, clinical features and tumor microenvironment among different subtypes of LUAD**. **(A)** Consensus clustering matrix at optimal k = 2. **(B)** Principal component analysis (PCA) between cluster1 and cluster2. **(C)** Kaplan-Meier curve of overall survival (OS) time for Cluster 1 and Cluster 2 (p = 0.013). **(D)** The heatmap of the variability of clinical features between subtypes. **(E)** GSEA analysis of two clusters. **(F)** The violin plot of differential infiltration of immune cells between cluster 1 and cluster 2. **(G)** The boxplot of the difference in ESTIMATE scores between the two subgroups. **(H)** The heatmap showed the differential expression of 28 immune checkpoint genes in cluster 1 and cluster 2.

Based on consensus clustering analysis, we divided the patients in the TCGA-LUAD cohort into two clusters (Clusters 1 and 2) based on the expression levels of the 15 m^6^A/m^1^A/A-to-I/APA-associated lncRNAs with prognostic value. Optimal clustering stability and least crossover between the LUAD samples were observed when the consensus matrix k value was equal to 2 (Figure 3A). PCA and T-SNE was performed to characterize the typed samples and the samples were visually distinguishable (Figure 3B and Supplementary Figure S1A). K-M survival curves constructed by “survival” and “survminer” revealed that the OS of Cluster 1 was worse than in Cluster 2 (p = 0.013) (Figure 3C). The heatmap showed that most of the above 15 prognostic lncRNAs were highly expressed in cluster 2 subgroup. Moreover, clinical correlation analysis showed that two clinical features, nodal metastasis status (N) and sex, were statistically different across subtypes, with Cluster 1 mostly comprising men and patients with lymph node metastases, whereas Cluster 2 included women and patients without lymph node metastases (Figure 3D). The GSEA results revealed that the pathways in Cluster 2 were primarily enriched in proteasomes, oxidative phosphorylation, and glyoxylate and dicarboxylate metabolism. The functional enrichment pathways for Cluster 1 were mainly enriched in the phosphatidylinositol signaling system, GnRH signaling pathway, and nitrogen metabolism (Figure 3E).

To investigate the effect of m^6^A/m^1^A/A-to-I/APA-associated prognostic lncRNAs on the immune microenvironment of patients with LUAD, we examined the differential levels of immune cell infiltration and TME score between Clusters 1 and 2 using the CIBERSORT and ESTIMATE algorithms, respectively. We found that resting CD4^+^ memory T cells and resting mast cells infiltrated notably less in Cluster 1 than in Cluster 2 (p<0.001), whereas activated mast cells, activated neutrophils, and activated CD4^+^ memory T cells infiltrated more in Cluster 1 than in Cluster 2 (p=0.002; p=0.012; p=0.012) (Figure 3F). Box diagrams of statistically significant immunocyte infiltration in the subgroups are shown in Supplementary Figure S1B. Compared with Cluster 1, the average immuneScore, stromalScore, and ESTIMATE Score were higher in Cluster 2 (Figure 3G, Supplementary Figure S1C, D).

We then analyzed the mRNA levels of immune checkpoint genes in each subtype and found that 15 genes were significantly different between the two subtypes. HHLA2, TNFSF14, VSIR, CD27, CD40LG, NCR3, BTLA, ENTPD1, CTLA4, and ICOS expression were lower in Cluster 1 than in Cluster 2, whereas the expression levels of TNFSF9, TNFSF4, TNFRSF18, FGL1, and CD276 were higher in Cluster 1 (Figure 3H).

These results indicated that these two subtypes were identified based on the expression of 15 prognostic RNA modification writer-related lncRNAs that differed significantly in terms of prognosis, clinicopathological features, and immune microenvironment. Such differences may be used to predict the different immunotherapeutic responses between the subgroups.

### 3.4 Construction of a risk prognostic signature by RNA modification writers-related lncRNAs in LUAD patients

**Figure 4:**
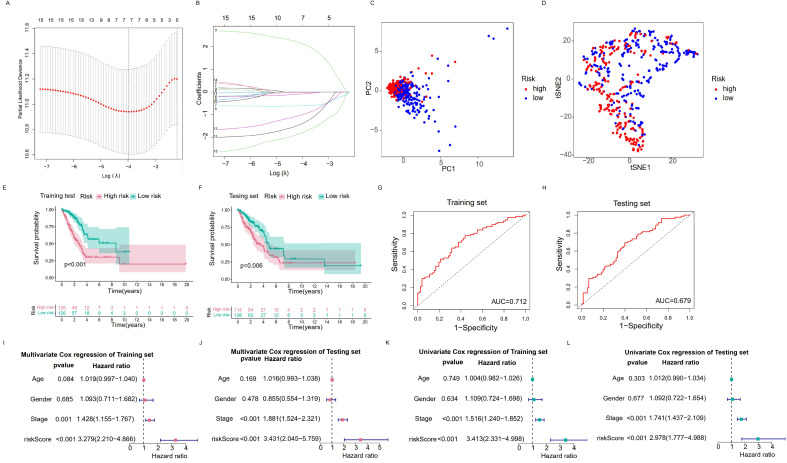
**The RNA modification writers-related lncRNAs prognostic signature. (A, B)** Least absolute shrinkage and selection operator (LASSO) cox regression for lncRNAs with prognostic value. **(C, D)** Principal component analysis (PCA) and t-distributed stochastic neighbor embedding (T-SNE) analysis of high risk group and low risk group. **(E, F)** Kaplan-Meier curves of OS of training set and testing set. **(G, H)** ROC curves of predicting 3-year survival for patients in the training and testing sets, the AUCs of the training and testing sets were 0.712 and 0.679, respectively. **(I, J)** Multivariate Cox regression analysis for the training and testing sets. **(K, L)** Univariate Cox regression analysis for the training and testing sets.

**Figure 5:**
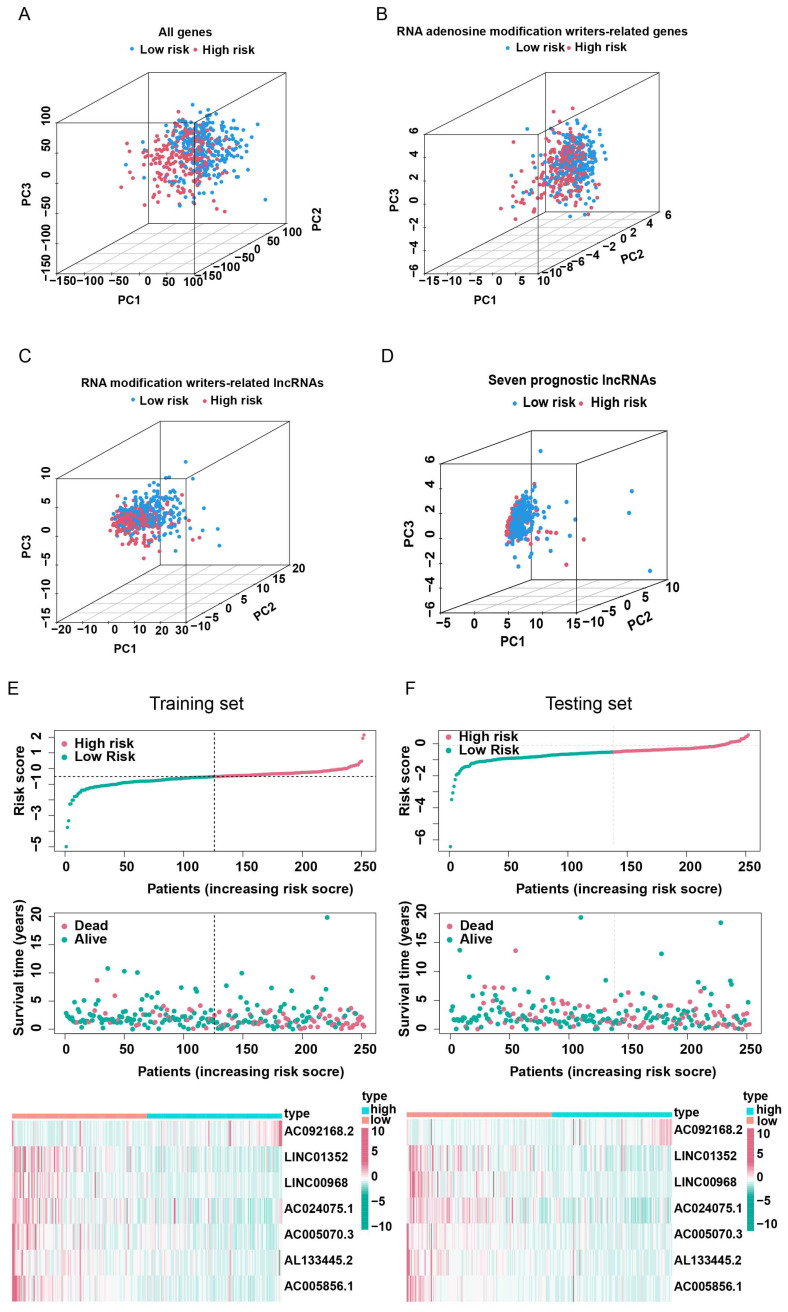
**Validation of lncRNAs-based prognostic risk models in training set and testing set.** Principal component analysis (PCA) based on the expression of **(A)** all genes, **(B)** RNA modification writers-related genes, **(C)** four types of RNA modification writers-related lncRNAs and (D) seven lncRNAs that constitute the prognostic signature. **(E, F)** Risk score curves, survival state distribution and expression heatmap of RNA modification writers-related lncRNAs in the training and testing sets.

In order to select the most prognostically significant lncRNAs and build a model predicting the prognosis of LUAD patients, Lasso Cox regression analysis was carried out based on the above 15 prognostic RNA modification writers-related lncRNAs**.** The partial likelihood deviance of the prognostic model is shown in Figure 4A, and the coefficients for these lncRNAs are shown in Figure 4B and Table 1. Seven lncRNAs (AC092168.2, LINC01352, LINC00968, AC024075.1, AC005070.3, AL133445.2, and AC005856.1) were included in our model construction using the minimum lambda criterion, and their coefficients were used to obtain the risk score. The calculation formula was as follows: risk score = (2.1239 * AC092168.2 expression) + (-0.6041 * LINC01352 expression) + (-0.0807 * LINC00968 expression) + (-0.0210 * AC024075.1 expression) + (-0.9854 * AC005070.3 expression) + (-1.3688 * AL133445.2 expression) + ( -1.777 * AC005856.1 expression). The TCGA-LUAD cohort was randomly divided into two sets in a 1:1 ratio, named as the training set and the test set, respectively, for risk model construction and validation. And the total TCGA -LUAD cohort was defined as the “entire set”. Based on the median risk score, the patients were divided into high- and low-risk subgroups. PCA and t-SNE analysis showed that the two risk subgroups were well-distinguished across the entire set (Figure 4C, D). K-M survival curves showed that the overall survival was worse for patients in the high-risk group than for those in the low-risk group, whether in the training subset (p < 0.001) or the validation subset (p=0.006) (Figure 4E, F), with an AUC of 0.712 and 0.679, respectively (Figure 4G, H). Univariate and multivariate Cox regression analyses showed that both clinical stage characteristics and risk scores could serve as independent prognostic factors in the training subset (p < 0.01) (Figure 4I, J). This conclusion was confirmed in the validation subset (Figure 4K, L). These results demonstrated that RNA modification writer-related lncRNAs can predict the prognosis of LUAD patients. In addition, based on PCA, no significant differences in gene expression were found between the two risk subgroups for all genes (Figure 5A), RNA modification writer-related genes (Figure 5B), or RNA modification writer-related lncRNAs (Figure 5C). In contrast, the expression of seven lncRNAs used to construct the prognostic model showed remarkable differences between the high- and low-risk groups (Figure 5D).

Furthermore, the distribution map of the risk scores and survival status in the training and testing subsets showed that patients in the high-risk subgroup had a worse prognosis than those in the low-risk subgroup. The expression levels of AC092168.2 were higher in the high-risk group, whereas protective m^6^A/m^1^A/A-I/APA-related lncRNAs, including LINC01352, LINC00968, AC024075.1, AC005070.3, AL133445.2, and AC005856.1, were lower in the high-risk group in both the LUAD training and testing sets (Figure 5E, F)**.**

The above results indicated that the prognostic model consisting of seven RNA modification writers-related lncRNAs could well predict the prognosis of LUAD patients. LUAD patients in the high-risk group have a worse prognosis than those in the low-risk group.

### 3.5 Risk prognostic signature was associated with clinical characteristics

**Figure 6:**
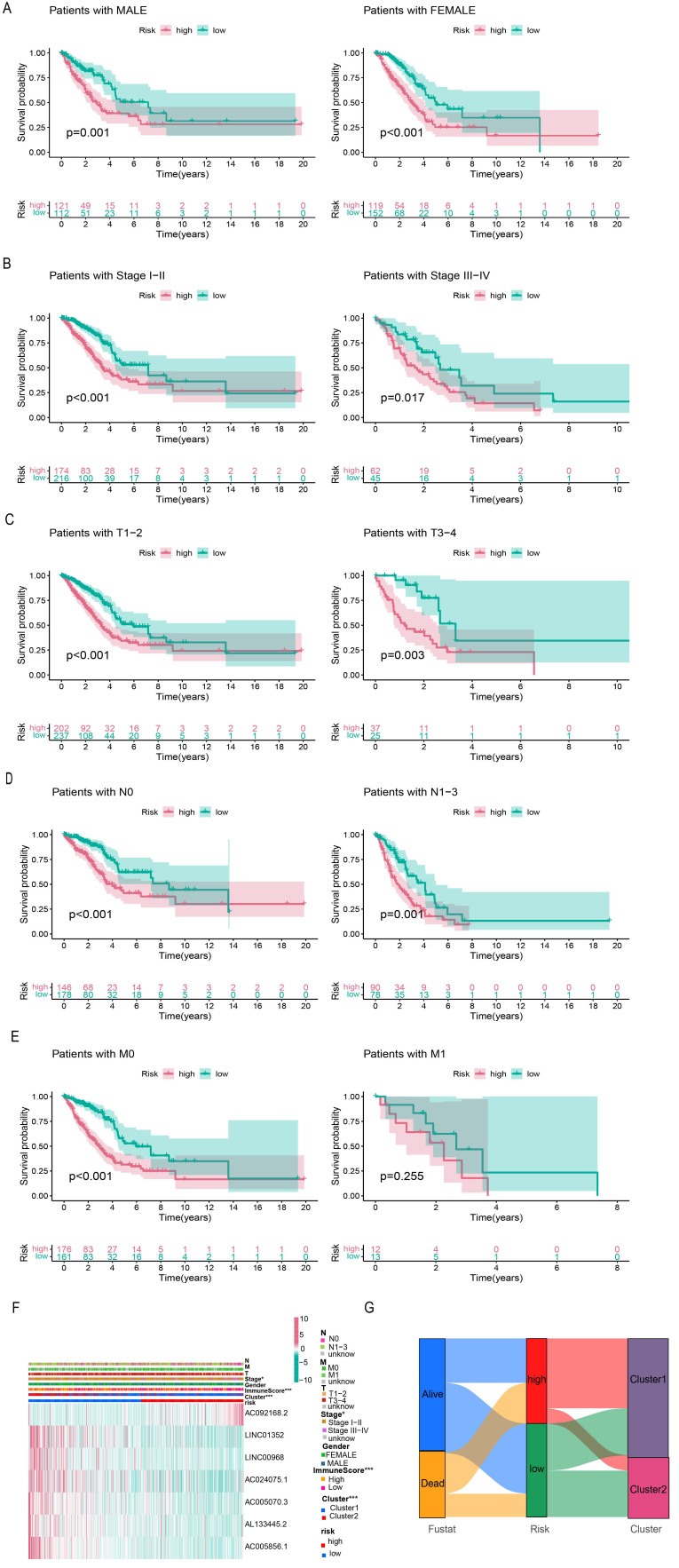
** Correlation analysis of risk signature subgroups with different clinical characteristics and OS of stratification based on clinical characteristics. (A-E)** Kaplan-Meier curves for overall survival of patients stratified by clinical characteristics in the high-risk and low-risk groups. **(F)** The heatmap of correlation between seven prognostic lncRNAs expression levels and clinicopathological features. **(G)** The Sankey diagram of the relationship between survival status, risk score and clustering typing.

The entire set was stratified into low- and high-risk patients based on the median risk score. According to different clinical characteristics, LUAD patients were sequentially classified into different strata: male and female; stage I-II and stage III-IV; T1-2 and T3-4; N0 and N1-3; M0 and M1. Then the differences in OS between the high- and low-risk subgroups in each stratum were analyzed. OS was better for low-risk patients than for high-risk patients in both sexes (Figure 6A). In addition, the prognostic model could predict the OS of LUAD patients at different TNM stages, except for M1 (limited by an inadequate sample size). The K-M survival curves showed that patients in the high-risk subgroup had a poorer prognosis than those in the low-risk subgroup (Figure 6B-E).

The heatmap displays the differential expression of the seven selected lncRNAs in high- and low-risk patients. Furthermore, differences in clinical stages, immune scores, and cluster subtypes were observed between the high- and low-risk groups. More specifically, patients in the early clinical stages were mostly distributed in the low-risk group, patients with low immune scores were mostly distributed in the high-risk group, and most patients in cluster 1 belonged to the high-risk group (Figure 6F). These findings were verified using a Sankey diagram (Figure 6G).

**Figure 7:**
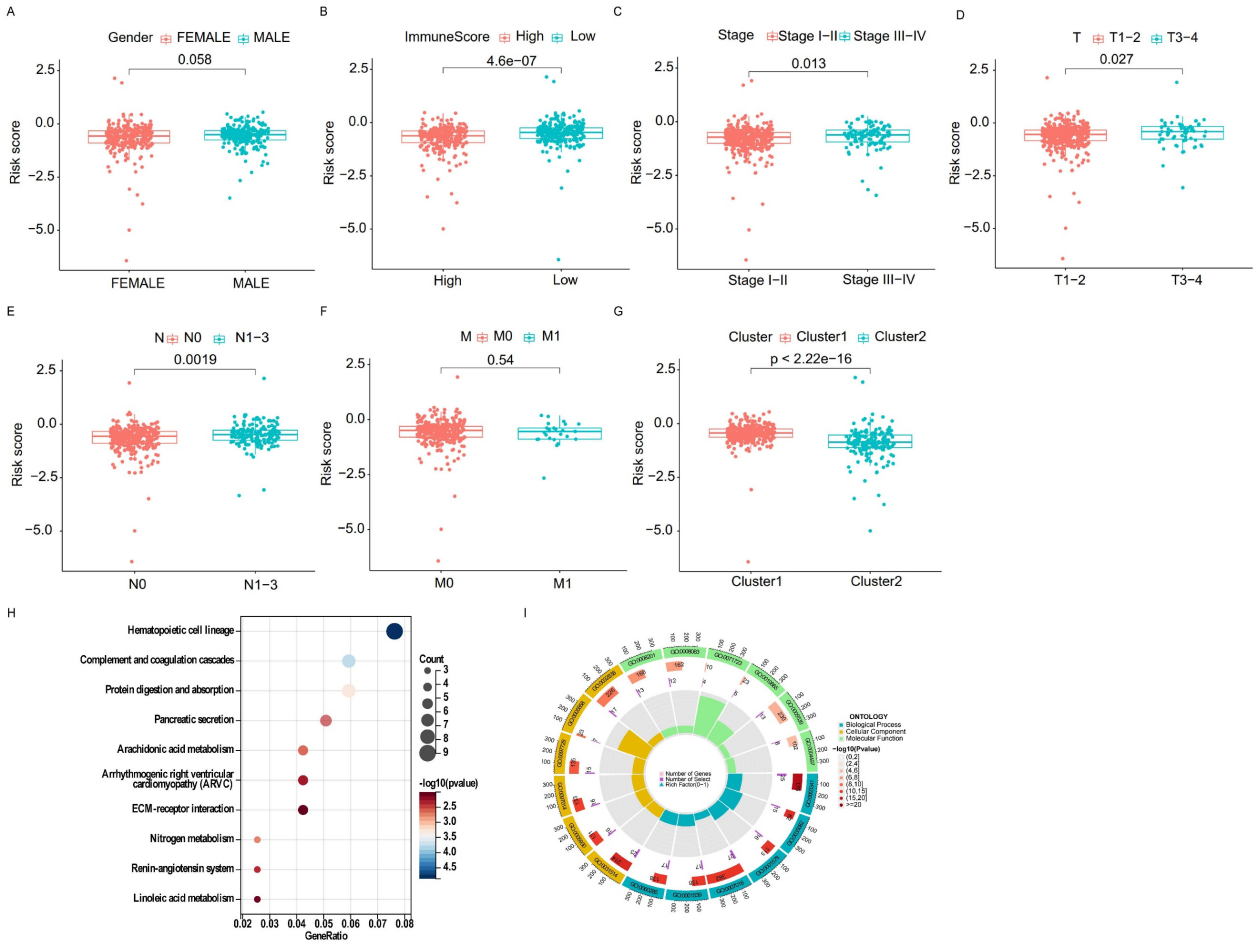
**Correlation of different clinical characteristics with risk scores. (A-G)** Differential analysis of risk scores for subgroups stratified based on clinicopathological characteristics. **(H, I)** GO and KEGG analysis.

By comparing the risk scores of patients stratified by different clinical characteristics, we found that patients in Cluster 1 had higher risk levels than those in Cluster 2, and patients with lower immune scores had higher risk scores than those with higher immune scores (Figure 7A, B). In addition, the risk increased with clinical stage, T-stage, and N-stage (all p-values ≤ 0.05); however, no difference was observed in M-stage (limited by the absence of information on M1 stage) or sex (Figure 7C-G).

Furthermore, the GO analysis revealed that genes in the high- and low-risk groups were enriched to the following molecular functions: heparin binding, growth factor activity, lipopeptide binding, immunoglobulin binding and glycosaminoglycan binding. (Figure 7H). KEGG pathway analysis showed that the DEGs between the high- and low-risk groups belonged to the following signaling pathways: hematopoietic cell lineage, ECM receptor interaction, and the renin-angiotensin system (Figure 7I). Taken together, these results showed that the clinicopathological grading was higher in the high-risk group compared to the low-risk group.

### 3.6 Analysis of the relevance of the m^6^A/m^1^A/A-to-I/APA model to the tumor immune microenvironment

**Figure 8:**
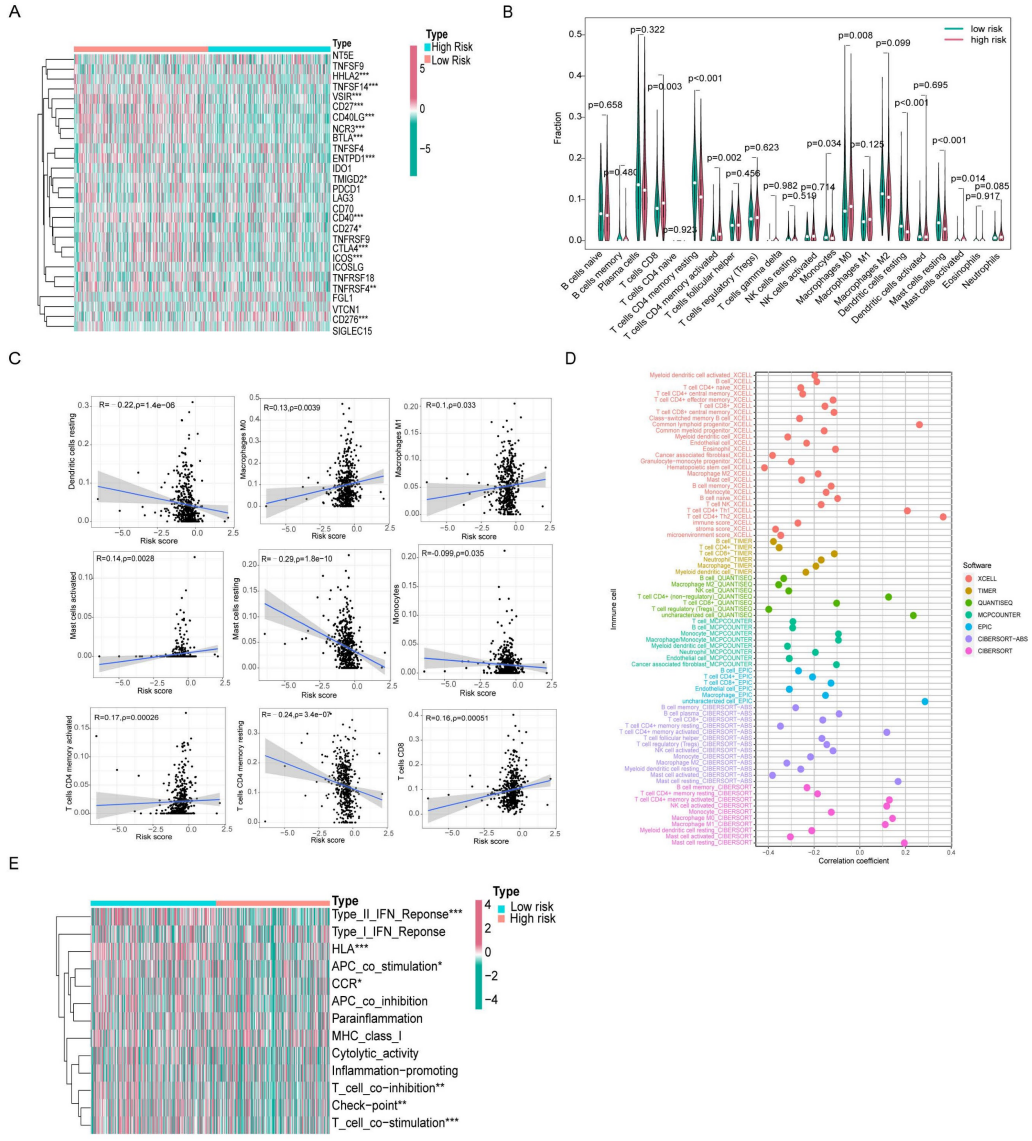
** Differences in immune microenvironment between high- and low-risk score groups. (A)** The heatmap showed differential expression of 28 immune checkpoint genes in the high- and low-risk groups. **(B)** The violin plot of differential infiltration of immune cells between different risk subgroups. **(C)** Scatter plots display of statistically significant correlation between immune infiltrating cells and risk scores. **(D)** Seven algorithms for quantifying the tumor microenvironment assessed the correlation between tumor microenvironment infiltrating cells and risk scores. **(E)** The heatmap demonstrated the differences in immune function between high and low risk groups.

We found 15 genes with different expression levels in the high- and low-risk groups, including HHLA2, TNFSF14, VSIR, CD27, CD40LG, NCR3, BTLA, ENTPD1, CD40, CD274, CTLA4, ICOS, TNFRSF4, TMIGD2, and CD276 by analyzing different immune checkpoint gene expression. In particular, HHLA2, TNFSF14, VSIR, CD27, CD40LG, NCR3, BTLA, ENTPD1, CD40, CD274, CTLA4, ICOS, TMIGD2, and TNFRSF4 were all highly expressed in the low-risk group, with the exception of CD276, which was highly expressed in the high-risk group. Ten of these genes overlapped in Cluster 2 and the low-risk groups (Figure 8A).

We used the CIBERSORT algorithm to calculate immune cell infiltration in different patients and created violin plots showing the differences between the high- and low-risk groups. CD8^+^ T cells, CD4^+^ activated memory T cells, macrophages M0, and mast cells activated had a higher level of infiltration in the high-risk group. In contrast, resting CD4^+^ memory T cells, monocytes, resting dendritic cells, and resting mast cells infiltrated less in the high-risk group (Figure 8B). Subsequently, we performed a relevance analysis of the risk score and immune cell infiltration. Figure 8C shows the infiltration levels of CD8^+^ T cells, M0 macrophages, M1 macrophages, activated mast cells, and activated CD4^+^ memory T cells, and the risk score was consistent, whereas the infiltration levels of resting CD4^+^ memory T cells, monocytes, resting dendritic cells, and resting mast cells and the risk score were opposite. For further comprehensive analysis, we used different algorithms, including xCELL, Timer, Quantiseq, MCPcounter, EPIC, CIBERSORT-ABS, and CIBERSORT, to assess cellular infiltration in the tumor microenvironment using the TCGA database. Bubble plots showed that hematopoietic stem cells, cancer-associated fibroblasts, and myeloid dendritic cells were negatively correlated with the risk score, whereas CD4^+^ Th1 T cells, CD4^+^ Th2 T cells, and common lymphoid progenitors were positively associated with the risk score (Figure 8D).

Furthermore, ssGSEA immune function analysis indicated that several pathways involved in immune function, such as the Type II IFN Response, HLA, APC co-stimulation, CCR, T cell co-inhibition, checkpoint, and T cell co-stimulation, were notably activated in the low-risk group (Figure 8E). The differences in immune checkpoint genes and immune cell infiltration in different risk groups reflect the potential value of prognostic models in predicting response to immunotherapy.

### 3.7 Analysis of gene mutations and comparison of the sensitivity of antitumor drugs

**Figure 9:**
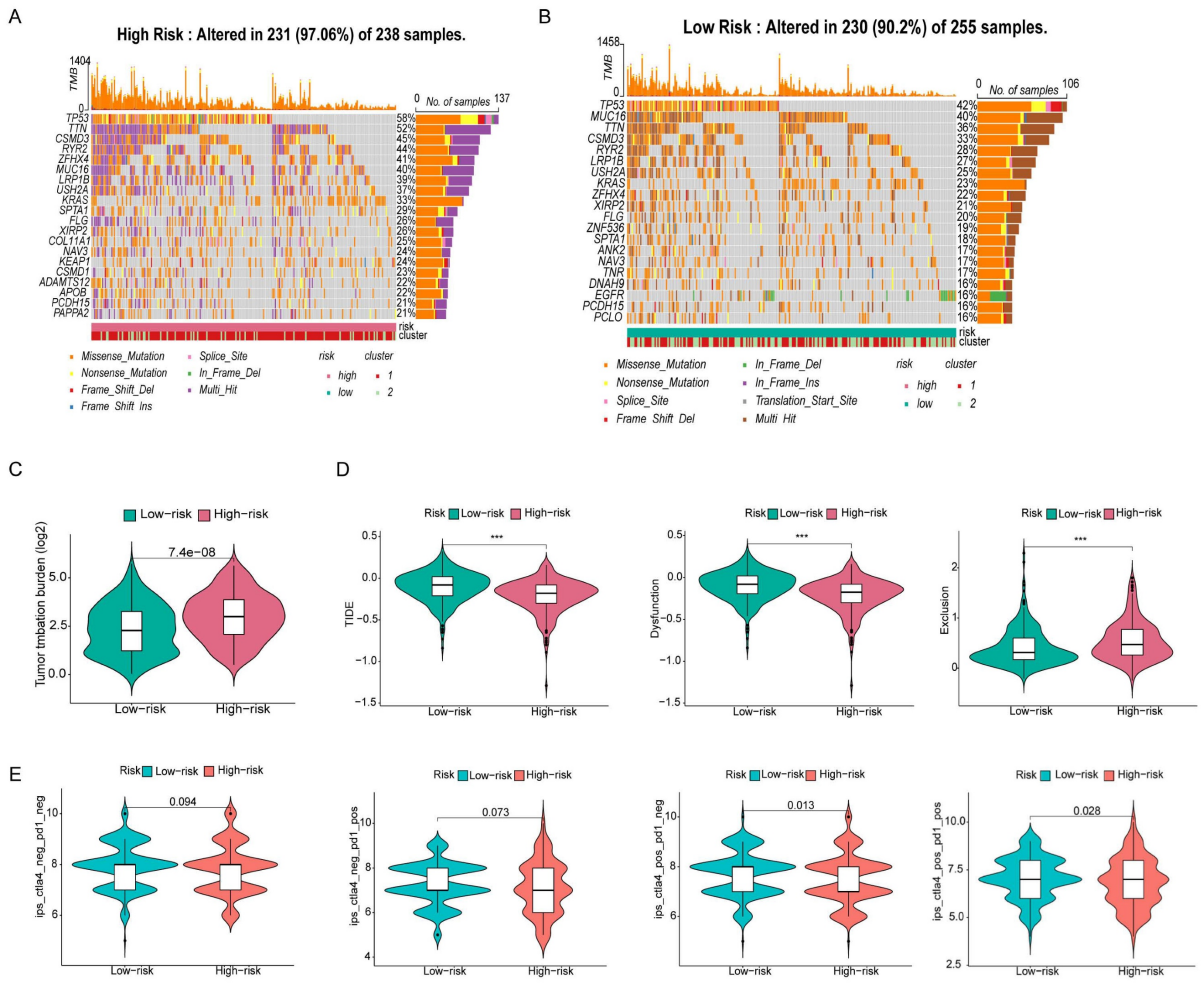
** Gene mutation analysis and immunotherapy response predictability in the prognostic signature. (A, B)** Waterfall plots of tumor mutation burden (TMB) for high- and low-risk groups, showing the top 20 genes with the highest mutation frequency. **(C)** Differences in TMB of patients with LUAD in high- and low-risk groups. **(D)** Differences in TIDE prediction scores between high- and low-risk groups (including TIDE score, dysfunction score, exclusion score). **(E)** Differences in IPS of patients with LUAD in high- and low-risk groups.

Because the predictive performance of TMB for the OS rate has been reported 31, we analyzed somatic mutations in the high- and low-risk groups separately and screened the top 20 genes with the greatest mutation incidences. TP53 showed the highest mutation rate in both subgroups (Figure 9A, B). Next, we compared the TMB differences between the high- and low-risk subgroups and found that the high-risk group had a higher tumor mutation burden (Figure 9C). TIDE can predict the response of patients treated with first-line anti-PD1 or anti-CTLA4 therapy and is therefore a novel predictive marker for immunotherapy 32. Patients with higher TIDE scores are likely to have a lower response rate to immunotherapy because their tumor cells are more likely to escape the immune system. Our results (Figure 9D) showed that the low-risk subgroup had a higher TIDE score, suggesting that it was less sensitive to immunotherapy. In addition, the effect of immunogenicity-based IPS in predicting the response to immunotherapy in melanoma patients has been reported 33. Figure 9E shows that the group with low-risk scores responded better to anti-PD-1 treatment (p=0.013), whereas the group with high-risk scores responded better to combination treatment with anti-PD-1 and CTLA-4 blockade (p=0.028). The high-risk group had higher TMB scores and lower TIDE scores, suggesting that the high-risk group was perhaps more sensitive to immunotherapy than the low-risk group.

**Figure 10:**
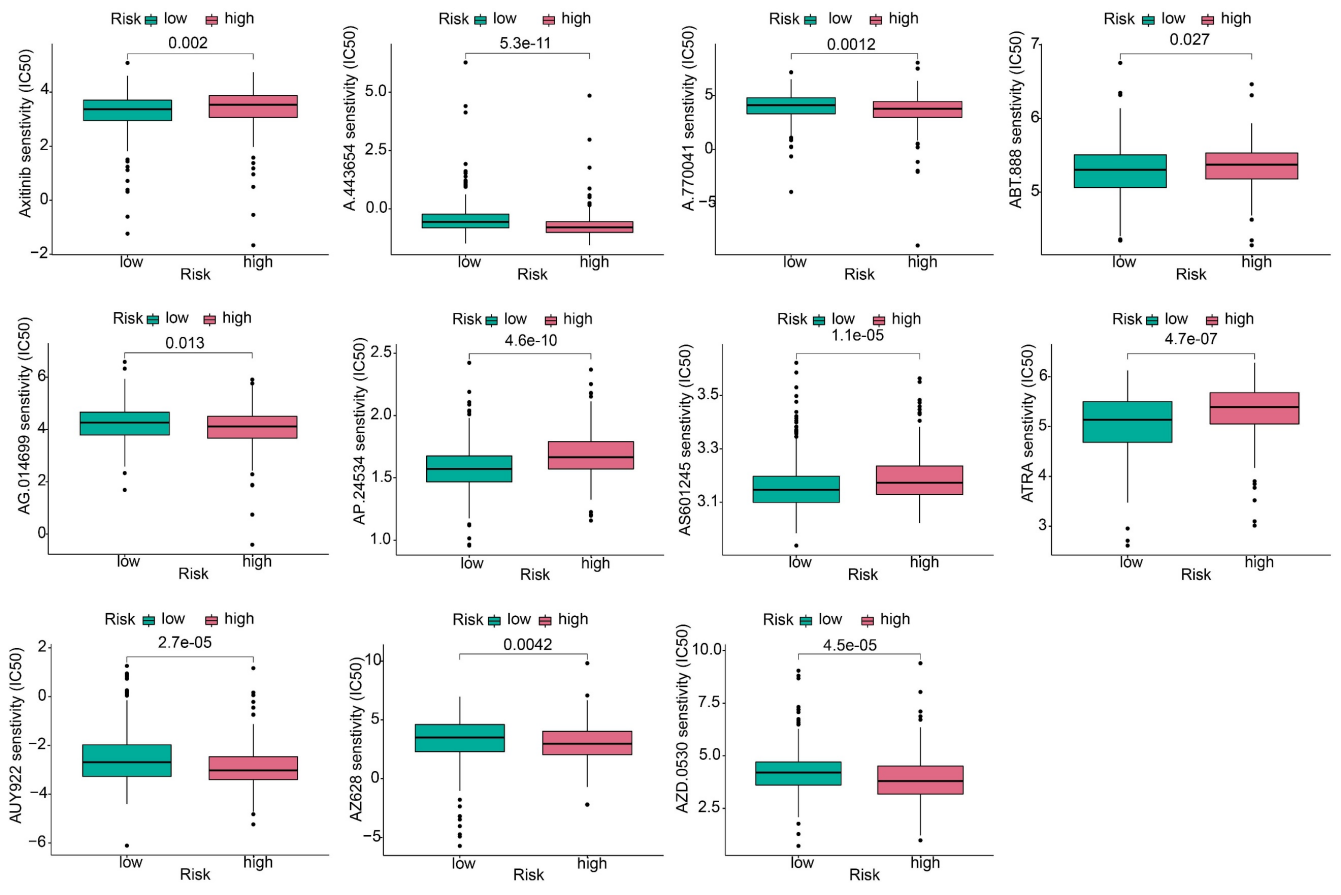
** Antitumor drug sensitivity analysis.** Comparison of the sensitivity of antitumor drugs in high and low risk groups.

To explore the effect of the risk score on anticancer drug therapy, the half-maximal inhibitory concentrations (IC_50_) of common drugs were compared between the two groups. The results showed that the IC_50_ values of osimertinib, ABT-888, AP.24534, AS601245, and ATRA were higher in the high-risk group, suggesting that patients in the low-risk group were more sensitive to these drugs. In contrast, A-443654, A.770041, AG.014699, AUY922, AZ628, and AZD.0530 exhibited the highest IC_50_ values in the low-risk group. The IC_50_ values of these drugs suggested that they were more effective in the high-risk group (Figure 10).

### 3.8 Prognostic value of RNA modification writers-related lncRNA in tumor microenvironment

**Figure 11:**
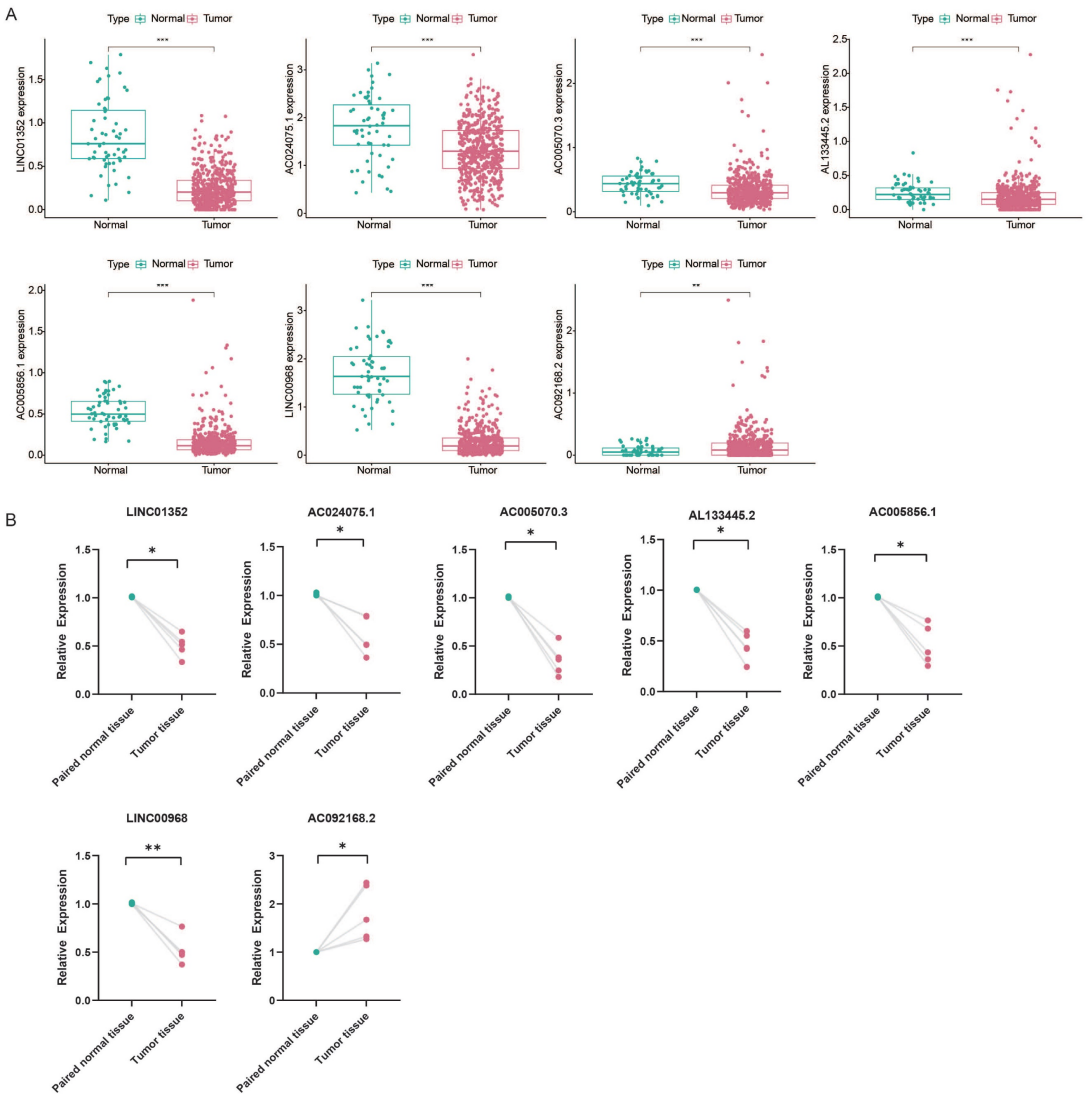

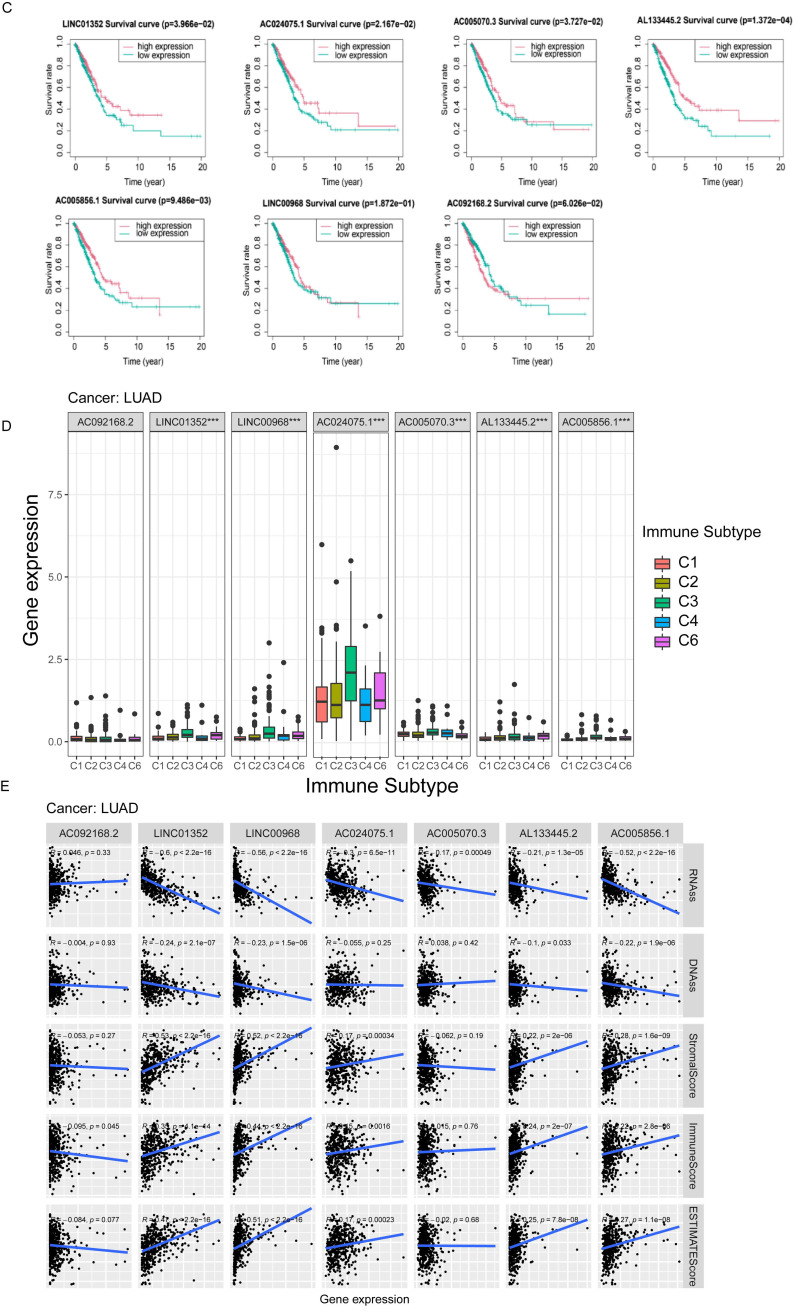
** Kaplan-Meier curves of overall survival and correlation analysis with immune response, TME and stemness score for each of the seven lncRNAs of risk signature. (A)** The expression of lncRNAs in TCGA database. **(B)** The expression of lncRNAs in paired LUAD patient samples by qPCR.** (C)** The correlation between the expression level of AC005070.3, AC005856.1, AC024075.1, AC092168.2, AL133445.2, LINC00968 and LINC01352 in the TCGA database and patient prognosis. **(D)** The correlation between lncRNA expression levels with prognostic value and immune subtypes in patients with lung adenocarcinoma. **(E)** Correlation of prognostic lncRNAs expression with TME (stromal score, immune score and estimation score) and stemness score (including DNAss and RNAss).

To explore the prognostic value of RNA modification writer-related lncRNAs in LUAD, we assessed their expression levels. Analysis of the TCGA database showed that LINC01352, AC024075.1, AC005070.3, AL133445.2, AC005856.1, and LINC00968 were downregulated in LUAD, whereas AC092168.2 was upregulated (Figure 11A). Furthermore, we examined the levels of these lncRNAs in paired samples obtained from LUAD patients. The qPCR results were consistent with the TCGA data (Figure 11B).

Based on the median expression value of each prognosis-related lncRNA, patients were divided into two groups: high and low expression. K-M survival curves showed that the expression levels of LINC01352, AC024075.1, AC005070.3, AL133445.2, and AC005856.1 in patients were associated with better OS (p < 0.05) (Figure 11C).

The results of the immune infiltration analysis demonstrate that high expression of LINC01352, LINC00968, AC024075.1, AC005070.3, AL133445.2, and AC005856.1 is correlated with the infiltration of C3 and C6 immune subtypes (Figure 11D). Additionally, we investigated the correlation between each lncRNA and the TME score. Figure 11E shows that LINC01352, LINC00968, AC024075.1, AL133445.2, and AC005856.1 were positively correlated with stromal score, immune core, and estimate core. Progressive loss of the differentiation phenotype and acquisition of the progenitor stem cell phenotype are important features in the development of cancer, and the stemness score is a new stem cell indicator for assessing the degree of tumor dedifferentiation 34. Therefore, we assessed the correlation of each lncRNA with RNAss, which reflected the gene expression profile of stem cells, and DNAss, which reflected the epigenetic profile of stem cells. As shown in Figure 11E, LINC01352, LINC00968, AC024075.1, AC005070.3, AL133445.2, and AC005856.1 were negatively correlated with RNAss, and LINC01352, LINC00968, AL133445.2, and AC005856.1 were negatively correlated with DNAss. These results suggested that LINC01352, AC024075.1, AC005070.3, AL133445.2 and AC005856.1 could act as protective factors in LUAD patients.

## 4. Discussion

Aberrant RNA methylation promotes tumor progression and immune regulation 35, and lncRNAs play critical roles in tumor metastasis and drug resistance by regulating gene expression 9. However, the correlation between RNA modifications and lncRNAs has not been fully elucidated 36, 37. In this study, seven lncRNAs associated with RNA modification writers were used to build a prognostic signature for predicting the OS rate of LUAD patients. In addition, patients categorized into high- and low-risk groups based on this signature showed differential immune checkpoint gene expression and tumor microenvironment cell infiltration patterns. These findings could help identify new prognostic markers and guide personalized therapies.

We first screened 269 lncRNAs from the TCGA-LUAD cohort, containing 504 tumor samples and 58 normal samples, for co-expression with four types of RNA modification “writer” enzymes, and further identified 15 lncRNAs with potential prognostic significance by univariate regression analysis. Based on the expression levels of these 15 lncRNAs, we used consensus clustering to group the samples into two potential subtypes, with a better OS rate in Cluster 2 than in Cluster 1. We further selected 7 of the 15 RNA modification writers-associated lncRNAs to construct a prognostic signature using the LASSO Cox regression method. Based on their correlation coefficients and expression levels, we obtained risk scores for each patient, whose median value allowed the samples to be classified into high- and low-risk subgroups with different OS rates. The Sankey diagram showing the relationships between risk scores, clusters, and survival status showed that patients in Cluster 1 were mostly distributed in the high-risk subgroup, whereas patients in the low-risk subgroup were mostly from Cluster 2. In both the training and validation sets, patients in the high-risk group had a worse prognosis than those in the low-risk group, which also suggested that both cluster typing based on 15 lncRNAs and a prognostic signature constructed based on seven lncRNAs had promising predictive potential for patients with LUAD. Both univariate and multivariate Cox regression analyses indicated that the risk score and clinical staging could serve as independent prognostic factors for LUAD patients. In addition, we validated the predictive power of the risk score in patients stratified according to clinicopathological parameters. Clinical correlation analysis showed that the risk scores were positively correlated with the clinical, T-stage, and N stages.

Our prognostic signature consisted of seven lncRNAs: AC092168.2, LINC01352, LINC00968, AC024075.1, AC005070.3, AL133445.2, and AC005856.1. Wu *et al.* built an immune-related prognostic signature, which also included AC092168.2, and revealed that it was strongly correlated with the prognosis of lung adenocarcinoma 36. HBx inhibits the expression of LINC01352, which increases the expression of miR-135b, thereby reducing adenomatous polyposis (APC) production and further activating the Wnt/β-catenin signaling pathway, promoting the progression of HBV-associated hepatocellular carcinoma 38. Record *et al.* constructed a ferroptosis-related lncRNA prognostic signature, including LINC01352, to predict the prognosis of lung adenocarcinoma 39. These results are consistent with our findings that LINC01352 is a protective factor against cancer, which has been validated in several studies. LINC00968 is heterogeneous and plays different roles in various cancers. In particular, it inhibits the progression of lung adenocarcinoma 40 and attenuates drug resistance in breast cancer 41; however, LINC00968 promotes epithelial ovarian cancer 42 and osteosarcoma 43. Nevertheless, the other lncRNAs in our signature panel have not yet been identified. We believe that our work will promote further investigation of the role of these RNA modification writer-related lncRNAs in tumor progression.

Immune-infiltrating cells constitute a major proportion of tumor stromal cells and are responsible for cancer development 26. RNA modifications and lncRNAs play active roles in immune cell infiltration and antitumor immune response 44, 45. Therefore, we explored the differences in immune-infiltrating cells, immune checkpoint gene expression, and TME scores between the different subtypes. Our results revealed that the immune scores, stromal scores and ESTIMATE scores in group 2 and the low-risk group were higher than those in group 1 and the high-risk group (Supplementary Figure S1E). In addition, the combined multi-algorithm integrated analysis of immune cell infiltration in relation to the risk score showed that the risk score was negatively correlated with most immune cell infiltrations. The ssGSEA results demonstrated a higher enrichment of immune-related pathways in the low-risk group.

Compared to classical cancer treatments such as surgery, chemotherapy, and radiotherapy, immunotherapy is a recently developed approach that has shown promising effects. Among the various immunotherapeutic strategies, immune checkpoint blockage is considered one of the most effective treatments for many types of cancers 46. In this study, we found that several common immune checkpoint-related genes were differentially expressed between high- and low-risk groups. In particular, HHLA2, TNFSF14, VSIR, CD27, CD40LG, NCR3, BTLA, ENTPD1, CD40, CD274, CTLA4, ICOS, TMIGD2, and TNFRSF4 were all highly expressed in the low-risk group, and most of these genes were more highly expressed in Cluster 2 than in Cluster 1. The immune checkpoint genes, including HERV-H LTR-associated protein 2 (HHLA2) 47, programmed cell death 1 ligand 1 (CD274/PD-L1) 48, cytotoxic T-lymphocyte-associated protein 4 (CTLA4) 49, inducible co-stimulatory factor (ICOS) 50 all belong to the B7-CD28 family, and the first three genes are suppressive immune checkpoints. CTLA4 and PD-L1 inhibitors are effective in cancer treatment 49. Therefore, HHLA2 is a potential target for cancer immunotherapy 47. ICOS has dual roles: it is a co-stimulatory receptor responsible for enhanced T cell responses to foreign antigens; in contrast, it promotes the immunosuppressive activity of Tregs and therefore exhibits pro-tumor activity 50. TNFSF14 51, CD27 52, CD40, and CD40LG 53 of the TNF superfamily are stimulatory immune checkpoints and their targeting may improve the efficacy of immunotherapy.

TMB and TIDE are predictive biological markers of immunotherapy 31, 54. The TMB and TIDE algorithms predict that patients in the high-risk group may benefit more from immune checkpoint blockade (ICB), possibly because of the complex TME and its pleiotropy in the low-risk group, where infiltration of immunosuppressive cells and expression of suppressive immune checkpoints assist tumor cells in gaining immune escape.

Owing to the diversity in patient sensitivity to drugs, it is necessary to personalize drug treatments for different patients. Therefore, we performed a sensitivity analysis of common antitumor drugs in different risk subgroups. The results indicated that ABT-888, AP.24534, AS601245, and ATRA were more effective in low-risk patients, whereas A-443654, A.770041, AG.014699, AUY922, and AZ628 were more suitable in high-risk patients.

Most of the seven m^6^A/m^1^A/A-to-I/APA-related lncRNAs had an HR >1 for LUAD patients, thereby acting as protective factors. This prompted us to further explore the prognostic value of the expression profile of each lncRNA. We found that the five lncRNAs were associated with significantly higher OS in the high-expression group than in the low-expression group based on the median expression level, which demonstrated that they were protective factors.

However, our study had certain limitations. First, owing to the limited number of LUAD samples in the TCGA database, a larger dataset needs to be included to validate our m^6^A/m^1^A/A-to-I/APA modification writer-related lncRNA model. Second, several types of RNA modifications, such as m^7^G, m^5^C, ac^4^C are also involved in tumor progression. The association between these RNA modifications and lncRNAs in cancer requires further investigation. In addition, RNA modifications not only depend on writer enzymes but also on other regulators such as erasers and readers, which requires further validation.

## 5. Conclusions

In summary, we established a risk model using four types of RNA modification writer-related lncRNAs to predict the prognosis of LUAD and revealed the association of this model with the tumor microenvironment and immunotherapy response of LUAD. This model may be useful for personalized therapy for LUAD patients.

## Supplementary Material

Supplementary figure and tables.

## Figures and Tables

**Table 1:** Gene coefficients

Gene	Coefficient
AC092168.2	2.12389597858716
LINC01352	-0.604139640810565
LINC00968	-0.0806516681426186
AC024075.1	-0.0210146370337467
AC005070.3	-0.985434252364944
AL133445.2	-1.36875743747402
AC005856.1	-1.77741270225117

## References

[1] Siegel RL, Miller KD, Fuchs HE, Jemal A (2022). Cancer statistics, 2022. CA-Cancer J Clin.

[2] Herbst RS, Morgensztern D, Boshoff C (2018). The biology and management of non-small cell lung cancer. Nature.

[3] Allemani C, Matsuda T, Di Carlo V, Harewood R, Matz M, Nikšić M (2018). Global surveillance of trends in cancer survival 2000-14 (CONCORD-3): analysis of individual records for 37513025 patients diagnosed with one of 18 cancers from 322 population-based registries in 71 countries. Lancet.

[4] Barbieri I, Kouzarides T (2020). Role of RNA modifications in cancer. Nat Rev Cancer.

[5] Li S, Mason CE (2014). The Pivotal Regulatory Landscape of RNA Modifications. Annu Rev Genomics Hum Genet.

[6] Gilbert WV, Bell TA, Schaening C (2016). Messenger RNA modifications: Form, distribution, and function. Science.

[7] Chen H, Yao J, Bao R, Dong Y, Zhang T, Du Y (2021). Cross-talk of four types of RNA modification writers defines tumor microenvironment and pharmacogenomic landscape in colorectal cancer. Mol Cancer.

[8] Ye L, Pan K, Fang S, Wu SN, Chen S, Tang S (2022). Four Types of RNA Modification Writer-Related lncRNAs Are Effective Predictors of Prognosis and Immunotherapy Response in Serous Ovarian Carcinoma. Front Immunol.

[9] Wen SM, Wei YL, Zen C, Xiong W, Niu YJ, Zhao Y (2020). Long non-coding RNA NEAT1 promotes bone metastasis of prostate cancer through N6-methyladenosine. Mol Cancer.

[10] Zou YT, Zheng SQ, Xie XH, Ye F, Hu XQ, Tian Z (2022). N6-methyladenosine regulated FGFR4 attenuates ferroptotic cell death in recalcitrant HER2-positive breast cancer. Nat Commun.

[11] Yin H, Chen L, Piao SQ, Wang YR, Li ZG, Lin Y (2023). M6A RNA methylation-mediated RMRP stability renders proliferation and progression of non-small cell lung cancer through regulating TGFBR1/SMAD2/SMAD3 pathway. Cell Death Differ.

[12] Dominissini D, Nachtergaele S, Moshitch-Moshkovitz S, Peer E, Kol N, Ben-Haim MS (2016). The dynamic N-1-methyladenosine methylome in eukaryotic messenger RNA. Nature.

[13] Shi Q, Xue C, Yuan X, He Y, Yu Z (2020). Gene signatures and prognostic values of m1A-related regulatory genes in hepatocellular carcinoma. Sci Rep.

[14] Zhao YS, Zhao QJ, Kaboli PJ, Shen J, Li MX, Wu X (2019). m1A Regulated Genes Modulate PI3K/AKT/mTOR and ErbB Pathways in Gastrointestinal Cancer. Transl Oncol.

[15] Tian B, Manley JL (2017). Alternative polyadenylation of mRNA precursors. Nat Rev Mol Cell Biol.

[16] Nishikura K (2016). A-to-I editing of coding and non-coding RNAs by ADARs. Nat Rev Mol Cell Biol.

[17] Molinie B, Wang JK, Lim KS, Hillebrand R, Lu ZX, Van Wittenberghe N (2016). m(6)A-LAIC-seq reveals the census and complexity of the m(6)A epitranscriptome. Nat Methods.

[18] Xiang JF, Yang Q, Liu CX, Wu M, Chen LL, Yang L (2018). N-6-Methyladenosines Modulate A-to-I RNA Editing. Mol Cell.

[19] Zhang MR, Song JM, Yuan WT, Zhang W, Sun ZQ (2021). Roles of RNA Methylation on Tumor Immunity and Clinical Implications. Front Immunol.

[20] Xue L, Li J, Lin Y, Liu D, Yang Q, Jian J (2021). m(6) A transferase METTL3-induced lncRNA ABHD11-AS1 promotes the Warburg effect of non-small-cell lung cancer. J Cell Physiol.

[21] Zhu S, Wang JZ, Chen D, He YT, Meng N, Chen M (2020). An oncopeptide regulates m(6)A recognition by the m(6)A reader IGF2BP1 and tumorigenesis. Nat Commun.

[22] Wilkerson MD, Hayes DN (2010). ConsensusClusterPlus: a class discovery tool with confidence assessments and item tracking. Bioinformatics.

[23] Lakota K, Thallinger GG, Sodin-Semrl S, Rozman B, Ambrozic A, Tomsic M (2012). International cohort study of 73 anti-Ku-positive patients: association of p70/p80 anti-Ku antibodies with joint/bone features and differentiation of disease populations by using principal-components analysis. Arthritis Res Ther.

[24] Nie J, Shan D, Li S, Zhang S, Zi X, Xing F (2021). A Novel Ferroptosis Related Gene Signature for Prognosis Prediction in Patients With Colon Cancer. Front Oncol.

[25] Newman AM, Liu CL, Green MR, Gentles AJ, Feng WG, Xu Y (2015). Robust enumeration of cell subsets from tissue expression profiles. Nat Methods.

[26] Yoshihara K, Shahmoradgoli M, Martinez E, Vegesna R, Kim H, Torres-Garcia W (2013). Inferring tumour purity and stromal and immune cell admixture from expression data. Nat Commun.

[27] Zhang CQ, Zhang Z, Zhang GC, Zhang ZH, Luo YJ, Wang F (2020). Clinical significance and inflammatory landscapes of a novel recurrence-associated immune signature in early-stage lung adenocarcinoma. Cancer Lett.

[28] Li Z, Wang W, Wu J, Ye X (2022). Identification of N7-methylguanosine related signature for prognosis and immunotherapy efficacy prediction in lung adenocarcinoma. Front Med (Lausanne).

[29] Fu JX, Li KR, Zhang WB, Wan CX, Zhang J, Jiang P (2020). Large-scale public data reuse to model immunotherapy response and resistance. Genome Med.

[30] Geeleher P, Cox N, Huang R (2014). Clinical drug response can be predicted using baseline gene expression levels and in vitro drug sensitivity in cell lines. J Invest Med.

[31] Ock CY, Hwang JE, Keam B, Kim SB, Shim JJ, Jang HJ (2017). Genomic landscape associated with potential response to anti-CTLA-4 treatment in cancers. Nat Commun.

[32] Jiang P, Gu SQ, Pan D, Fu JX, Sahu A, Hu XH (2018). Signatures of T cell dysfunction and exclusion predict cancer immunotherapy response. Nat Med.

[33] Charoentong P, Finotello F, Angelova M, Mayer C, Efremova M, Rieder D (2017). Pan-cancer Immunogenomic Analyses Reveal Genotype-Immunophenotype Relationships and Predictors of Response to Checkpoint Blockade. Cell Reports.

[34] Malta TM, Sokolov A, Gentles AJ, Burzykowski T, Poisson L, Weinstein JN (2018). Machine Learning Identifies Stemness Features Associated with Oncogenic Dedifferentiation. Cell.

[35] Haruehanroengra P, Zheng YY, Zhou YB, Huang Y, Sheng J (2020). RNA modifications and cancer. RNA Biol.

[36] Wu GM, Wang QH, Zhu T, Fu LH, Li ZP, Wu YL (2021). Identification and Validation of Immune-Related LncRNA Prognostic Signature for Lung Adenocarcinoma. Front Genet.

[37] Wang E, Li Y, Ming R, Wei J, Du P, Zhou P (2021). The Prognostic Value and Immune Landscapes of a m(6)A/m(5)C/m(1)A-Related LncRNAs Signature in Head and Neck Squamous Cell Carcinoma. Front Cell Dev Biol.

[38] Huang PB, Xu QD, Yan YC, Lu YJ, Hu ZG, Ou B (2020). HBx/ER alpha complex-mediated LINC01352 downregulation promotes HBV-related hepatocellular carcinoma via the miR-135b-APC axis. Oncogene.

[39] Tang H, Wu ZX, Zhang Y, Xia TT, Liu D, Cai JR (2019). Identification and Function Analysis of a Five-Long Noncoding RNA Prognostic Signature for Endometrial Cancer Patients. DNA Cell Biol.

[40] Tang HP, Han XL, Feng Y, Hao YQ (2020). linc00968 inhibits the tumorigenesis and metastasis of lung adenocarcinoma via serving as a ceRNA against miR-9-5p and increasing CPEB3. Aging-US.

[41] Xiu DH, Liu GF, Yu SN, Li LY, Zhao GQ, Liu L (2019). Long non-coding RNA LINC00968 attenuates drug resistance of breast cancer cells through inhibiting the Wnt2/beta-catenin signaling pathway by regulating WNT2. J Exp Clin Cancer Res.

[42] Yao N, Sun JQ, Yu L, Ma L, Guo BQ (2019). LINC00968 accelerates the progression of epithelial ovarian cancer via mediating the cell cycle progression. Eur Rev Med Pharmacol Sci.

[43] Liu G, Yuan DT, Sun P, Liu WD, Wu PF, Liu H (2018). LINC00968 functions as an oncogene in osteosarcoma by activating the PI3K/AKT/mTOR signaling. J Cell Physiol.

[44] Guo LT, Yang H, Zhou CF, Shi Y, Huang L, Zhang J (2021). N6-Methyladenosine RNA Modification in the Tumor Immune Microenvironment: Novel Implications for Immunotherapy. Front Immunol.

[45] Li X, Ma S, Deng Y, Yi P, Yu J (2022). Targeting the RNA m(6)A modification for cancer immunotherapy. Mol Cancer.

[46] Huang X, Zhang XZ, Li EL, Zhang G, Wang X, Tang TY (2020). VISTA: an immune regulatory protein checking tumor and immune cells in cancer immunotherapy. J Hematol Oncol.

[47] Bhatt RS, Berjis A, Konge JC, Mahoney KM, Klee AN, Freeman SS (2021). KIR3DL3 Is an Inhibitory Receptor for HHLA2 that Mediates an Alternative Immunoinhibitory for Pathway to PD1. Cancer Immunol Res.

[48] Alexander PG, McMillan DC, Park JH (2021). A meta-analysis of CD274 (PD-L1) assessment and prognosis in colorectal cancer and its role in predicting response to anti-PD-1 therapy. Crit Rev Oncol/Hematol.

[49] Liu JN, Kong XS, Huang T, Wang R, Li W, Chen QF (2020). Clinical Implications of Aberrant PD-1 and CTLA4 Expression for Cancer Immunity and Prognosis: A Pan-Cancer Study. Front Immunol.

[50] Amatore F, Gorvel L, Olive D (2018). Inducible Co-Stimulator (ICOS) as a potential therapeutic target for anti-cancer therapy. Expert Opin Ther Targets.

[51] Skeate JG, Otsmaa ME, Prins R, Fernandez DJ, Da Silva DM, Kast WM (2020). TNFSF14: LIGHTing the Way for Effective Cancer Immunotherapy. Front Immunol.

[52] Buchan SL, Rogel A, Al-Shamkhani A (2018). The immunobiology of CD27 and OX40 and their potential as targets for cancer immunotherapy. Blood.

[53] Aloui C, Prigent A, Tariket S, Sut C, Fagan J, Cognasse F (2016). Levels of human platelet-derived soluble CD40 ligand depend on haplotypes of CD40LG-CD40-ITGA2. Scientific Reports.

[54] Wang SX, He ZK, Wang X, Li HM, Liu XS (2019). Antigen presentation and tumor immunogenicity in cancer immunotherapy response prediction. eLife.

