# Supplementary material for: Prognostic Value and Immune Landscapes of Four Types of RNA Modification Writer-Related LncRNAs Signature in Lung Adenocarcinoma
Source: J Cancer. 2024 Jul 9;15(15):4818–37. doi: 10.7150/jca.96755 (PMC11310873; doi:10.7150/jca.96755)

Supplementary Table 1. Comparisons of the clusters' clinicopathological characteristics

	Type	Cluster1	Cluster2	P value
Gender	FEMALE	166(50.30%)	67(38.51%)	0.167
	MALE	164(49.70%)	107(61.49%)	
Stage	Stage I-II	246(74.55%)	144(82.76%)	0.2105
	Stage III-IV	79(23.94%)	28(16.09%)	
	unknow	5(1.52%)	2(1.15%)	
T stage	T1-2	283(85.76%)	156(89.66%)	0.3098
	T3-4	46(13.94%)	16(9.20%)	
	unknow	1(0.30%)	2(1.15%)	
M stage	M0	237(71.82%)	100(57.47%)	0.3461
	M1	19(5.76%)	6(3.45%)	
	unknow	74(22.42%)	68(39.08%)	
N stage	N0	196(59.40%)	128(73.56%)	0.1901
	N1-3	128(38.79%)	40(22.99%)	
	unknow	6(1.82%)	6(3.45%)	

Supplementary Table 2. Comparison of clinicopathological characteristics between two sets

Covariates	Type	Total	Testing Set	Training Set	P value
Age	<=65	238 (47.2%)	109 (43.2%)	129 (51.1%)	0.10
	>65	256 (50.8%)	137 (54.3%)	119 (47.2%)	
	unknow	10 (1.9%)	6 (2.4%)	4 (1.6%)	
Gender	Male	233 (46.2%)	110 (43.7%)	123 (48.8%)	0.28
	Female	271 (53.8%)	142 (56.4%)	129 (51.2%)	
Stage	Stage I	271 (53.8%)	140 (55.6%)	131 (52.0%)	0.18
	Stage II	119 (23.6%)	63 (25.0%)	56 (22.2%)	
	Stage III	81 (16.1%)	32 (12.7%)	49 (19.4%)	
	Stage IV	26 (5.2%)	15 (5.9%)	11 (4.4%)	
	unknow	7 (1.4%)	2 (0.8%)	5 (1.9%)	
T stage	T1	168 (33.3%)	82 (32.6%)	86 (34.1%)	0.62
	T2	271 (53.8%)	142 (56.4%)	129 (51.1%)	
	T3	43 (8.5%)	19 (7.5%)	24 (9.5%)	
	T4	19 (3.8%)	8 (3.2%)	11 (4.4%)	
	unknow	3 (0.6%)	1 (0.4%)	2 (0.7%)	
M stage	M0	337 (66.9%)	166 (65.9%)	171 (67.9%)	0.41
	M1	25 (4.9%)	15 (5.9%)	10 (4.0%)	
	unknow	142 (28.2%)	71 (28.2%)	71 (28.2%)	
N stage	N0	324 (64.3%)	163(64.7%)	161 (63.9%)	0.23
	N1	95 (18.9%)	52 (20.6%)	43 (17.1%)	
	N2	71 (14.1%)	30 (11.9%)	41 (16.3%)	
	N3	2 (0.4%)	0 (0.0%)	2 (0.8%)	
	NX	1 (0.2%)	1 (0.4%)	0 (0.0%)	
	unknow	11 (2.2%)	6 (2.4%)	5 (2.0%)	

Supplementary Table 3. PCR primer sequences.

Primers		Sequence (5'-3')
LINC01352	Forward	GGTTGATTGAAACTCTCCCTTCT
	Reverse	TGGTCCTACCTGAATTGGCT
AC092168.2	Forward	GACCTGAGCCATCAATCATCCA
	Reverse	GGGAGACACAAGAGAGAAACTAAGC
LINC00968	Forward	GCTGGGTAACTTCAGGTGGA
	Reverse	TGTCTTCCTGGTCCCAAACG
AC024075.1	Forward	AGAGATCGTGCTGCTGCTAA
	Reverse	GCCTCCTGAGTATCTGGGATTAC
AC005070.3	Forward	CTCAAGTCATCCTACCACCTTAGC
	Reverse	TGGGCAGCATAACGAGACC
AL133445.2	Forward	CCTACTGTGGTGTTGCGGATA
	Reverse	ATTTGGAAACAATGCTACTGGAGAG
AC005856.1	Forward	AATACCCACCCACCAGCAG
	Reverse	AAGTGTAACATTCATCAGCCTCTC

Supplementary Figure S1. (A) T-distributed stochastic neighbor embedding (T-SNE) analysis between cluster1 and cluster2. **(B)** The boxplot of differences in immune cell infiltration in two subgroups. **(C)** The boxplot of the difference in immune scores between the two subgroups. **(D)** The boxplot of the difference in stromal scores between the two subgroups. **(E)** Differences in TME scores (including immune score, stromal score and ESTIMATE score) across risk subgroups.

Supplementary Figure S1

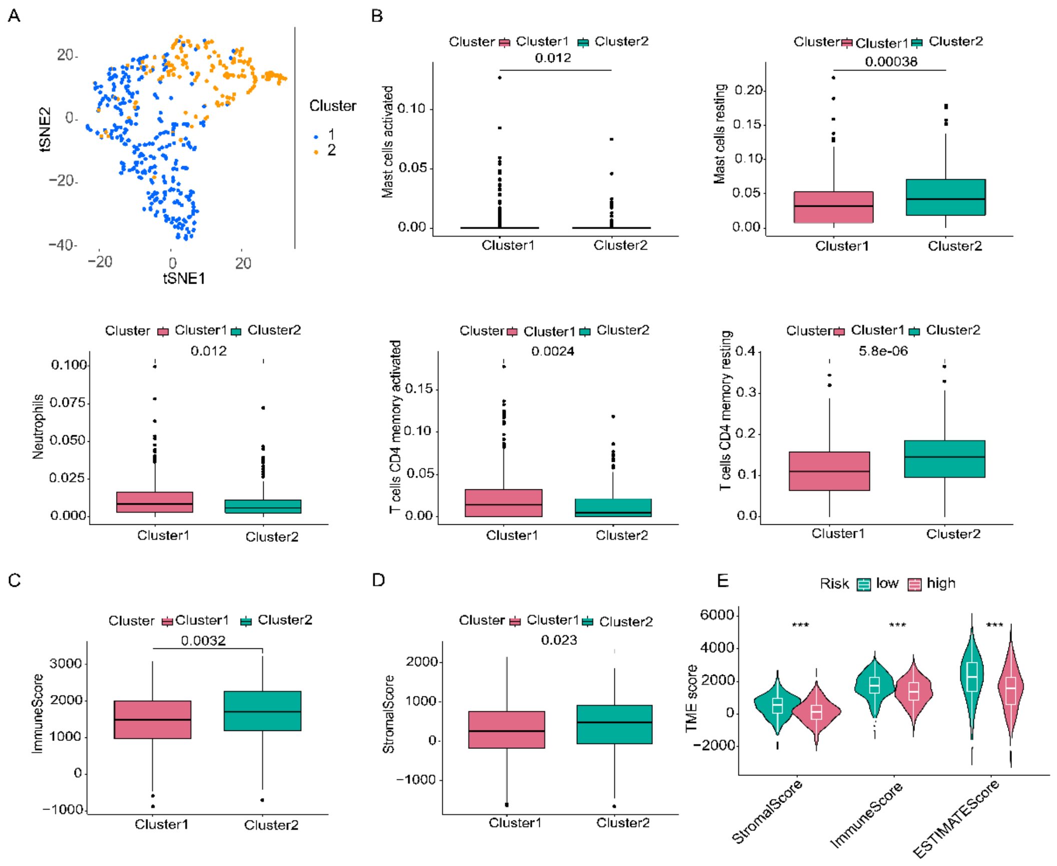

Supplement: Supplementary file 1 — Supplementary figure and tables. [file jcav15p4818s1.pdf]
